# Supplementary material for: In Silico and In Vitro Evaluation of Some Amidine Derivatives as Hit Compounds towards Development of Inhibitors against Coronavirus Diseases
Source: Viruses. 2023 May 15;15(5):1171. doi: 10.3390/v15051171 (PMC10223987; doi:10.3390/v15051171)
Supplement: Supplementary file 1 [file viruses-15-01171-s001.zip › viruses-2304140-supplementary.pdf]

Supplementary information

In Silico and In Vitro Evaluation of Some Amidine Derivatives as Hit Compounds towards Development of Inhibitors against Coronavirus Diseases

Ahmed H. E. Hassan [1,*], Selwan M. El-Sayed [1], Mizuki Yamamoto [2], Jin Gohda [2], Takehisa Matsumoto [3], Mikako Shirouzu [3], Jun-ichiro Inoue [4], Yasushi Kawaguchi [2,5], Reem M. A. Mansour [1], Abtin Anvari [6] and Abdelbasset A. Farahat [6,7,*]

[1] Department of Medicinal Chemistry, Faculty of Pharmacy, Mansoura University, Mansoura 35516, Egypt; salwanmahmoud@mans.edu.eg (S.M.E.-S.); reemmansour@std.mans.edu.eg (R.M.A.M.)
[2] Research Center for Asian Infectious Diseases, Institute of Medical Science, The University of Tokyo, Tokyo 108-8639, Japan; mizuyama@g.ecc.u-tokyo.ac.jp (M.Y.); jgohda@g.ecc.u-tokyo.ac.jp (J.G.); ykawagu@g.ecc.u-tokyo.ac.jp (Y.K.)
[3] Drug Discovery Structural Biology Platform Unit, RIKEN Center for Biosystems Dynamics Research, Kanagawa 230-0045, Japan; takehisa.matsumoto@riken.jp (T.M.); mikako.shirouzu@riken.jp (M.S.)
[4] Infection and Advanced Research Center (UTOPIA), The University of Tokyo Pandemic Preparedness, Tokyo 108-8639, Japan; jun-i@g.ecc.u-tokyo.ac.jp
[5] Division of Molecular Virology, Department of Microbiology and Immunology, The Institute of Medical Science, The University of Tokyo, Tokyo 108-8639, Japan
[6] Master of Pharmaceutical Sciences Program, California Northstate University, 9700 W Taron Dr., Elk Grove, CA 95757, USA; abtin.anvari8815@cnsu.edu
[7] Department of Pharmaceutical Organic Chemistry, Faculty of Pharmacy, Mansoura University, Mansoura 35516, Egypt
* Correspondence: ahmed_hassan@mans.edu.eg (A.H.E.H.); abdelbasset.farahat@cnsu.edu (A.A.F.)

1. In silico docking

The crystal structure of TMPRSS2 (PDB: 7MEQ) was downloaded from the protein databank. Protein preparation was performed using preparation wizard in MOE and solvent and non-protein residues other than the co-crystallized ligand were removed. Hydrogen atoms were added, and charges were calculated. Ligands to be investigated were sketched, prepared and their energies were minimized to generate a library database. Binding site was defined based on the co-crystallized ligand and docking simulations were run as implemented in MOE. The output was visualized and analysed considering binding with the catalytic triad residues and other amino acid residues within the binding site.

2. Chemistry

2.1 General

All commercial reagents were used without purification. Melting points were determined on a Mel-Temp 3.0 melting point apparatus and were uncorrected. TLC analysis was carried out on silica gel 60 F254 precoated aluminium sheets using a UV light for detection. ^{1}HNMR spectra were recorded on a Bruker 600 MHz spectrometer using the indicated solvents. High-resolution mass spectra were obtained from the Georgia State University Mass Spectrometry Laboratory, Atlanta, GA. Elemental analyses were performed by Atlantic Microlab Inc., Norcross, GA.

2.2 General procedure for the synthesis of compounds 8–10.

Sodium metabisulphite (0.75 g, 4 mmol) solution in water (2 ml) was added to a stirred solution of the bisbenzaldehyde derivative (2 mmol) in ethanol (20 mL) and stirring was continued for 1 h. 2-(3,4-diaminophenyl)-1*H*-benzo[d]imidazole-6-carboximidamide hydrochloride or 3,4-diamino-*N*-*iso*-propylbenzimidamide (4 mmol) was added to the reaction mixture and the mixture was refluxed for 24 h. The reaction mixture was concentrated under reduced pressure and filtered, the formed precipitate was suspended in water and neutralized with sodium hydroxide solution (2 N), filtered, and dried under vacuum at room temperature. The free base was dissolved in methanol (80 ml) and filtered. Finally, ethanolic HCl was added to the filtrate and stirred for 24 h. The solution was concentrated to 5 ml and filtered, washed with ethanol and acetone, and dried at 100°C under vacuum for 24 h.

2,2'-(Pyridine-2,6-diyl)bis(1*H*-benzo[d]imidazole-6-carboximidamide) (8).

Gray solid, yield (0.48 gN, 41%), mp >300°C; ^{1}HNMR (DMSO-d_6) δ 9.48 (s, 4H), 9.14 (s, 4H), 8.54 (d, 2H, J=7.8 Hz), 8.32 (m, 3H), 7.96 (d, 2H, J=8.4 Hz), 8.01 (d, 2H, J=8.4 Hz); ESI-MS: m/z calculated for $C_{21}H_{18}N_9$: 396.1685, found: 396.1679 (amidine base $[M+1]^+$); Anal. Calcd. For $C_{21}H_{17}N_9$. 4HCl. 2.5H_2O: C, 43.01; H, 4.46; N, 21.50. Found: C, 42.83; H, 4.49; N, 21.29.

2,2'-(Pyridine-2,6-diyl)bis(*N*-*iso*-propyl-1*H*-benzo[d]imidazole-6-carboximidamide) (9).

Green solid, yield (0.39 g, 25%), mp >300°C; ^{1}HNMR (DMSO-d_6) δ 9.61 (s, 2H), 9.47 (s, 3H), 9.06 (s, 3H), 8.44 (d, 2H, J=7.8 Hz), 8.27 (t, 1H, J=7.8 Hz), 8.15 (br s, 2H), 7.90 (d, 2H, J=7.8 Hz), 7.64 (br s, 2H), 4.11 (m, 2H), 1.33 (d, 12H, J=6.6 Hz); ESI-MS: m/z calculated for $C_{27}H_{30}N_9$: 480.2624, found: 480.2646 (amidine base $[M+1]^+$); Anal. Calcd. For $C_{27}H_{29}N_9$. 4HCl. $3H_2O$: C, 47.72; H, 5.78; N, 18.55. Found: C, 47.38; H, 5.71; N, 18.44.

2,2'-(1,3-Phenylene)bis(1*H*-benzo[d]imidazole-6-carboximidamide) (10).

Yellow solid, yield (0.24 g, 21%), mp >300°C; ^{1}HNMR (DMSO-d_6) δ 9.57 (s, 4H), 9.27 (s, 4H), 8.60 (d, 2H, J=7.8 Hz), 8.30 (m, 2H), 7.91 (m, 4H), 7.84 (d, 2H, J=7.8 Hz); ESI-MS: m/z calculated for $C_{22}H_{19}N_8$: 395.1733, found: 395.1729 (amidine base $[M+1]^+$); Anal. Calcd. For $C_{22}H_{18}N_8$. 4HCl. $3H_2O$: C, 44.46; H, 4.74; N, 18.85. Found: C, 44.39; H, 4.78; N, 18.49.

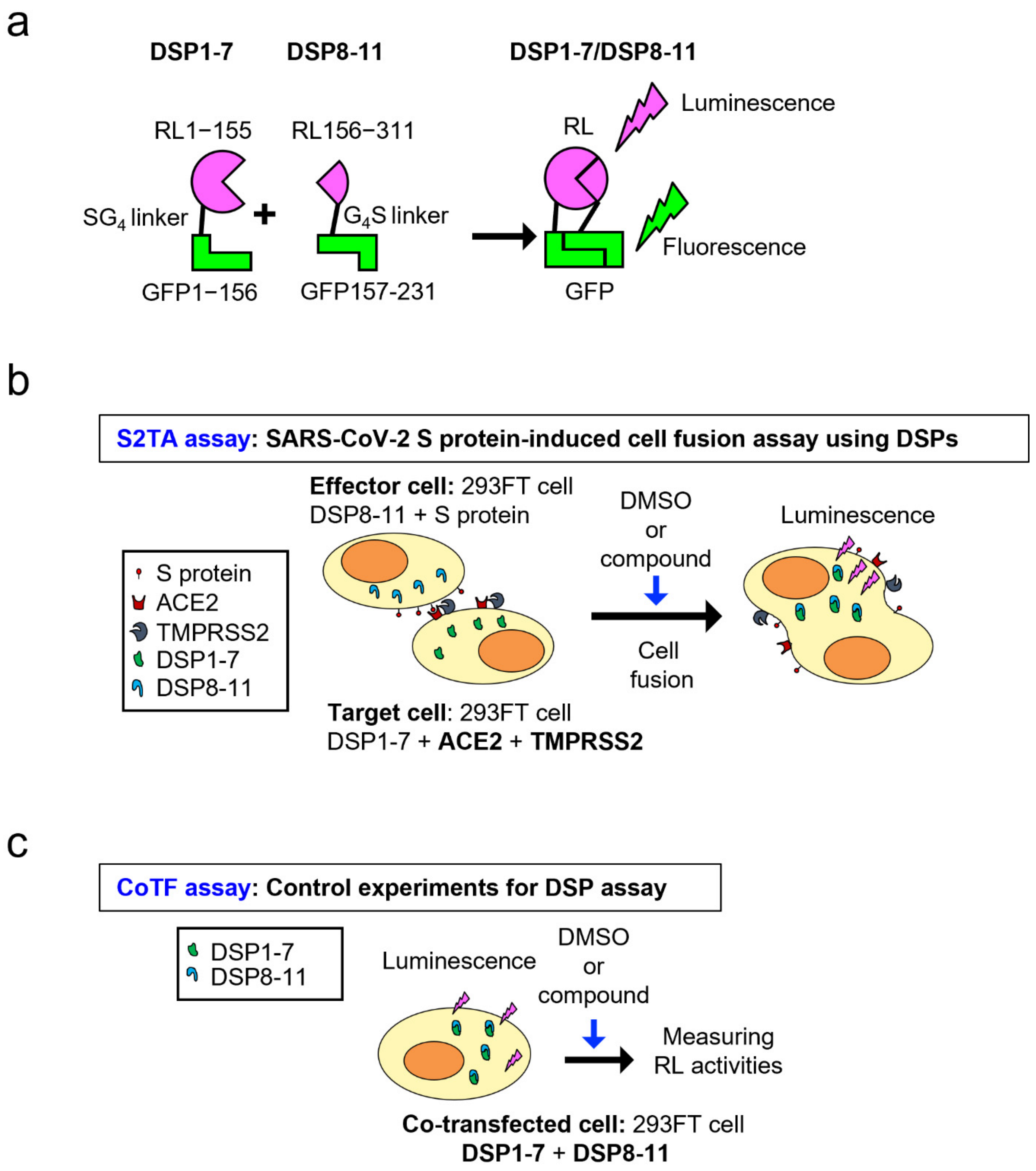

Supplementary Figure S1. Cell-based membrane fusion assay for SARS-Cov-2 S protein using the DSP reporter. (**a**) Schematic representation of split chimeric reporter proteins. DSP1-7 has the structure RL1-155-Ser-Gly-Gly-Gly-Gly-GFP1-156. DSP8-11 has the structure Met-GFP157-231-Gly-Gly-Gly-Gly-Ser-RL156-311. DSP1-7 and DSP8-11 efficiently reassociate, resulting in the reconstitution of functional RL and GFP to produce

luminescent and fluorescent signals, respectively. (**b**) A method for monitoring cell fusion mediated by the S protein of SARS-CoV-2. Effector cells (DSP8-11 and S protein expressing 293FT cells) and target cells (DSP1-7, ACE2 and TMPRSS2 expressing 293FT cells) were co-cultured for 4 h. Both GFP (fluorescence) and RL (luminescence) signals were generated following DSP1-7 and DSP8-11 reassociation upon mixing of the cells during this assay. (**c**) A method for testing whether compounds directly inhibit DSP activity without affecting cell fusion. DSP1-7 and DSP8-11 co-expressing 293FT cells were treated with compounds for 4 h. RL activities of the DSP1-7/DSP8-11 complex were measured to test whether the compounds directly inhibit RL activities without affecting cell fusion.

**Peptide substrate**		**Campany**
for thrombin	dabcyl-FSARGHRP-E(EDANS)-NH2	Peptide Institute
for factor Xa	dabcyl-IEGRTATS-E(EDANS)-NH2	Peptide Institute
for TMPRSS2	dabcyl-SSRQSRIVGG-E(EDANS)-NH2	GeneScript

**Enzyme**		**Company**
Thrombin	cat.1473-SE	R&D SYSTEMS
Factor Xa	cat.1063-SE	R&D SYSTEMS
TMPRSS2	baculovirus expression systems	Made in-house

Peptide Institute, Osaka, Japan
GenScript, NJ, USA
R&D Systems, MN, USA

Supplementary Figure S2. Enzymes and substrates for enzyme assays.
